# Exercise does not influence development of phenotype in *PLN* p.(Arg14del) cardiomyopathy

**DOI:** 10.1007/s12471-023-01800-4

**Published:** 2023-07-20

**Authors:** Freyja H. M. van Lint, Fahima Hassanzada, Tom E. Verstraelen, Weijia Wang, Laurens P. Bosman, Paul A. van der Zwaag, Toon Oomen, Hugh Calkins, Brittney Murray, Crystal Tichnell, Thais M. A. Beuren, Folkert W. Asselbergs, Arjan Houweling, Maarten P. van den Berg, Arthur A. M. Wilde, Cynthia A. James, J. Peter van Tintelen

**Affiliations:** 1 Department of Genetics, University Medical Centre Utrecht, Utrecht University, Utrecht, The Netherlands; 2 Department of Human Genetics, Amsterdam University Medical Centres, location Academic Medical Centre, University of Amsterdam, Amsterdam, The Netherlands; 3 Netherlands Heart Institute, Utrecht, The Netherlands; 4 Heart Centre, Department of Cardiology, Amsterdam University Medical Centre, location Academic Medical Centre, University of Amsterdam, Amsterdam, The Netherlands; 5 Division of Cardiology, Department of Medicine, Johns Hopkins University, Baltimore, MD USA; 6 Department of Cardiology, University Medical Centre Utrecht, Utrecht University, Utrecht, The Netherlands; 7 University Medical Centre Groningen, Department of Genetics, University of Groningen, Groningen, The Netherlands; 8 Department of Cardiology, St. Antonius Hospital, Sneek, The Netherlands; 9 Department of Cardiology, University Medical Centre Groningen, University of Groningen, Groningen, The Netherlands; 10 Amsterdam Cardiovascular Sciences, Heart Failure and Arrhythmias, Amsterdam, The Netherlands

**Keywords:** Arrhythmogenic right ventricular cardiomyopathy, Dilated cardiomyopathy, Genetics, Exercise, Penetrance

## Abstract

**Background:**

Endurance and frequent exercise are associated with earlier onset of arrhythmogenic right ventricular cardiomyopathy (ARVC) and ventricular arrhythmias (VA) in desmosomal gene variant carriers. Individuals with the pathogenic c.40_42del; p.(Arg14del) variant in the *PLN* gene are frequently diagnosed with ARVC or dilated cardiomyopathy (DCM). The aim of this study was to evaluate the effect of exercise in *PLN* p.(Arg14del) carriers.

**Methods:**

In total, 207 adult *PLN* p.(Arg14del) carriers (39.1% male; mean age 53 ± 15 years) were interviewed on their regular physical activity since the age of 10 years. The association of exercise with diagnosis of ARVC, DCM, sustained VA and hospitalisation for heart failure (HF) was studied.

**Results:**

Individuals participated in regular physical activities with a median of 1661 metabolic equivalent of task (MET) hours per year (31.9 MET-hours per week) until clinical presentation. The 50% most and least active individuals had a similar frequency of sustained VA (18.3% vs 18.4%; *p* = 0.974) and hospitalisation for HF (9.6% vs 8.7%; *p* = 0.827). There was no relationship between exercise and survival free from (incident) sustained VA (*p* = 0.65), hospitalisation for HF (*p* = 0.81), diagnosis of ARVC (*p* = 0.67) or DCM (*p* = 0.39) during follow-up. In multivariate analyses, exercise was not associated with sustained VA or HF hospitalisation during follow-up in this relatively not-active cohort.

**Conclusion:**

There was no association between the amount of exercise and the susceptibility to develop ARVC, DCM, VA or HF in *PLN* p.(Arg14del) carriers. This suggested unaffected *PLN* p.(Arg14del) carriers can safely perform mild-moderate exercise, in contrast to desmosomal variant carriers and ARVC patients.

**Supplementary Information:**

The online version of this article (10.1007/s12471-023-01800-4) contains supplementary material, which is available to authorized users.

## What’s new?

- In carriers of *PLN* p.(Arg14del), an exercise history of mild-moderate exercise prior to clinical presentation does not predict: (1) development of ventricular arrhythmias or heart failure (time/severity) or (2) a diagnosis of cardiomyopathy.
- There is no reason to limit mild-moderate exercise in *PLN* p.(Arg14del) individuals without signs or symptoms.
- As malignant ventricular arrhythmias occur in nearly 50% of these individuals while being active, a clinical cardiomyopathy diagnosis or the presence of arrhythmia-related risk factors (decreased left ventricular ejection fraction, prior sustained or non-sustained ventricular tachycardia, low-voltage electrocardiogram, number of leads with negative T waves or premature ventricular contraction count) warrant discussion of exercise restrictions.

## Introduction

Inherited cardiomyopathies, such as arrhythmogenic cardiomyopathy (ACM) and dilated cardiomyopathy (DCM), may present with arrhythmias, heart failure (HF), or even sudden cardiac death (SCD). ACM can present predominantly in the right ventricle (arrhythmogenic right ventricular cardiomyopathy (ARVC)), the left ventricle, or both. In ARVC patients, (likely) pathogenic variants are mainly identified in genes encoding desmosomal proteins [1]. In DCM patients, variants can be identified in a broad spectrum of genes, mainly encoding proteins of the cytoskeleton, sarcomere or nucleus. The non-desmosomal variant p.(Arg14del) in the gene encoding phospholamban (*PLN*) is common in the Netherlands and can be found both in patients diagnosed with ARVC or DCM [2–4]. *PLN* is important for Ca^2+^ homeostasis in cardiac muscle by regulating the sarcoplasmic reticulum Ca^2+^ pump [5]. Individuals with *PLN* p.(Arg14del) generally present from adulthood onwards with a low-voltage electrocardiogram (ECG), negative T waves, a high count of premature ventricular contractions (PVCs), malignant ventricular arrhythmias (VA) and HF [6]. Factors that promote penetrance and influence expression in this autosomal dominantly inherited disorder remain unknown [7].

Because several studies have confirmed a role for vigorous exercise in penetrance and arrhythmic risk in ARVC, guidelines recommend that individuals with ARVC should not participate in competitive or frequent high-intensity exercise [8]. High duration and intensive exercise have been demonstrated to increase penetrance among carriers of desmosomal variants and risk of developing a ventricular tachycardia (VT), structural dysfunction, and HF [9–13]. Even in genotype-positive family members exercise restriction was found to be protective [14]. However, exercise also gives physical and mental advantages [15], and restrictions prevent individuals from benefitting from these. As *PLN* p.(Arg14del) may manifest as ARVC [4], we evaluated the influence of exercise on age-related penetrance, arrhythmic risk, and progression to HF.

## Methods

### Study population

The study population was recruited from 3 Dutch university medical centres (Amsterdam, Groningen and Utrecht). Subjects who were a carrier of *PLN* p.(Arg14del) and ≥ 18 years of age were invited by a letter to participate in a detailed interview to document exercise history or could self-apply via the website of the PLN Patient Foundation (www.plnheartdiseasefoundation.org). The study protocol was exempted from approval by the Medical Ethics Committee of the Amsterdam University Medical Centres because the Act of Medical Research involving Human Subjects did not apply (W17_076 #17.094).

### Genotype and phenotype

Genotype was derived from sequencing of *PLN* in a diagnostic setting [2]. Clinical and demographic data were drawn from the ACM registry [16].

### Clinical variables

Clinical presentation was defined as a first visit for *PLN* p.(Arg14del) related complaints or family screening. We used 3 clinical endpoints: a clinical diagnosis of ARVC or DCM, sustained VA and hospitalisation for HF [17]. Sustained VA was defined as ventricular tachycardia of > 30 s or < 30 s when terminated electrically (including appropriate implantable cardioverter-defibrillator (ICD) therapy) or pharmacologically; ventricular fibrillation (VF); and/or resuscitated or aborted cardiac arrest, as described previously [7]. When an individual reached one of these clinical endpoints, he/she was defined as having a cardiac phenotype and considered penetrant for disease. ARVC diagnosis was assigned according to the 2010 Task Force Criteria [18]. DCM diagnosis was based on decreased left ventricular ejection fraction (LVEF) and increased left ventricular volume (LVEDD > 112% of predicted corrected for age and body surface area, and a reduced LV function [EF < 45% or fractional shortening < 35% on echo] or cardiac MR with LVEF < 45% and left ventricular end-diastolic volume > 2 SD above reference value) [17, 19]. Risk factors for malignant ventricular arrhythmia or HF, previously established in *PLN* p.(Arg14del), were also collected: LVEF < 45%, (non‑)sustained ventricular tachycardia (VT), > 500 PVC count/24 h, number of leads showing negative T leads and presence of low-voltage ECG [6, 7].

### Exercise interviews

Exercise history was obtained by structured telephone interviews. Participants were prompted to list regular exercise done since the age of 10 years for leisure including sports, for work, and for transportation (by bicycle). Details of collection of intensity and duration of each activity have previously been described [9]. All responses were transcribed on pre-prepared data collection sheets.

### Analysis of exercise history

For each participant, type and intensity of the activities were expressed in metabolic equivalent (MET) using the Ainsworth compendium [20] (FvL). This MET-score reflects the energy expenditure in comparison to a resting state [21]. By multiplying the frequency and duration of each activity with the MET-values, an estimation of annual MET-hours was obtained. We calculated exercise dose (MET-hours/year) preceding and following clinical presentation.

### Statistical analysis

Categorical variables are reported as frequency (%) and group differences were calculated by using the chi-square or Fisher’s exact test. Continuous variables are reported as mean ± standard deviation (SD) or median (interquartile range) and were compared across groups using a *t*-test or Mann-Whitney U test. MET-hours/year before and after clinical presentation were determined and compared using the Wilcoxon signed rank test. The cumulative probability of survival free from clinical endpoints was calculated with the Kaplan-Meier method. For individuals without a clinical outcome, end of follow-up (censoring) was defined by last clinical follow-up date.

Differences in survival between individuals when split by median or quartiles of annual MET-hours of activity were evaluated with the log-rank test. Cox proportional hazard models with a Firth correction for rare outcomes were used to test the association of exercise prior to clinical presentation and outcomes. Known risk factors for *PLN* p.(Arg14del) carriers were selected as covariates [6, 7]. These covariates at clinical presentation, were controlled for using different models based on the hypothetical relations between variables. Model 1 adjusts only for age and sex, whereas model 2 consists of all covariates. Missing values were accounted for by multiple imputation (R-package MICE; 20 imputation sets with each 10 iterations). Results were pooled over the imputation sets using Rubin’s rules. Restricted cubic spline analyses were performed to further assess the association between mean MET-hours/year until presentation as a continuous variable and risk of outcomes. A two-sided *p*-value of < 0.05 was considered significant. Statistical analyses were performed using SPSS (version 25.0; IBM Corp., Armonk, NY, USA), and RStudio (version 1.1.383; RStudio, Boston, MA, USA).

## Results

### Study population

Out of 590 adults from the PLN Registry (April 2017), 346 (58.6%) agreed to be interviewed, of whom 75 were self-referred via the PLN Foundation’s website. Of them, 299 (86.4%) were interviewed, of whom 207 (69.2%) had an adequate amount of clinical information available to determine phenotype and clinical outcomes with a median follow-up duration of 5.9 years.

Tab. 1 summarises the clinical characteristics. A minority were male (39.1%) and most presented because of family history (65.7%). At last follow-up 18% had experienced a sustained VA and more than 9% had been hospitalised for HF. Nine participants experienced both a sustained VA and hospitalisation for HF at last follow-up.

**Table 1:** Clinical characteristics of study population

Characteristic	Total (*N* = 207)	Active (*n* = 104)^a^	Inactive (*n* = 103)^b^	*P*-value
Male	81 (39.1)	47 (45.2)	34 (33.0)	0.064
Proband	48 (23.2)	24 (23.1)	24 (23.3)	0.608
Caucasian	207 (100)	104 (100)	103 (100)	1
Age at interview, years	53 ± 15	53 ± 13	52 ± 16	0.707
Mean MET-hours per year until presentation	1661 (852–3199)	3199 (1662–5623)	852 (381–1190)	< 0.001
*Presentation*				
Age at presentation, years	45 ± 15	45 ± 14	44 ± 16	0.576
Type of presentation:				
– Resuscitated SCD	4 (1.9)	4 (3.8)	0	0.121
– Symptomatic	48 (23.2)	23 (22.1)	25 (24.3)	0.713
– Sustained VA	13 (6.3)	8 (7.7)	5 (4.9)	0.241
– Positive family history	136 (65.7)	66 (63.5)	70 (68.0)	0.495
*At last follow-up visit*				
Age at last clinical follow-up, years	52 ± 15	52 ± 13	51 ± 16	0.716
Task Force Criteria met	19 (9.2)	10 (9.6)	9 (8.7)	0.259
Fulfilment DCM diagnosis	52 (25.1)	28 (26.9)	24 (23.3)	0.193
ICD implantation	72 (34.8)	38 (36.5)	34 (33.0)	0.594
Sustained VA:	38 (18.4)	19 (18.3)	19 (18.4)	0.974
– Sustained VT/VF	18 (8.7)	11 (10.6)	7 (6.8)	0.334
– Appropriate ICD shock	15 (7.2)	7 (6.7)	8 (7.8)	0.774
Hospitalisation for heart failure	19 (9.2)	10 (9.6)	9 (8.7)	0.827
RVEF < 45%	39 (30.2)	24 (34.3)	15 (25.4)	0.275
LVEF < 45%	49 (23.7)	28 (35)	21 (29.2)	0.442
Cardiac transplantation	11 (5.3)	7 (6.7)	4 (3.9)	0.834
Death	2 (1)	0	2 (1.9)	0.224
Follow-up duration, years	5.9 (2.8–9.1)	6 (3.1–8.8)	5.6 (2.3–9)	0.692

*SCD* sudden cardiac death, *VA* ventricular arrhythmias, *DCM* dilated cardiomyopathy, *VT* ventricular tachycardia, *VF* ventricular fibrillation, *ICD* implantable cardioverter-defibrillator, *RVEF* right ventricular ejection fraction, *LVEF* left ventricular ejection fraction

Data are *n* (%), mean ± standard deviation, or median (interquartile range)

^a^Active group consisted of individuals who participated in 50% highest mean metabolic equivalent of task (*MET*) hours per year until presentation

^b^Inactive group consisted of individuals who participated in 50% lowest mean MET-hours per year until presentation

### Exercise history

Exercise history before and after clinical presentation are shown in Tab. 2. As can be appreciated, this was not a particularly athletic cohort. Prior to presentation, the study population participated in activities with a median of 1661 MET-hours/year, which included leisure activities, such as sports, with a median of 490 MET-hours/year (9.4 MET-hours/week). This equals walking for pleasure for 2.5 h per week. The Dutch Health Council advises adults to exercise at a moderate intensity for half an hour 5 days per week at a minimum [22], which is met by half of the Dutch population [23].

**Table 2:** Exercise history of study population before and after clinical presentation (*N* = 207)

Time spent on activity	Before presentation	After presentation	*P-*value
Mean MET-hours/year spent on any activity^a^	1661 (852–3200)	1152 (199–2880)	< 0.001
– Leisure activity	490 (168–1148)	282 (0–720)	< 0.001
– Work activity	607 (0–2239)	0 (0–2095)	0.004
– Transportation activity	0 (0–203)	0	< 0.001
Mean hours/year spent on any activity	367 (147–1038)	180 (36–840)	< 0.001
– Leisure activity	79 (28–162)	45 (0–137)	0.002
– Work activity	178 (0–850)	0 (0–720)	0.028
– Transportation activity	0 (0–49)	0	< 0.001
Assigned METs of activities performed	4.1 (3.1–5.2)	3.4 (2.6–5.0)	< 0.001

Data median (interquartile range)

^a^Metabolic equivalent of task (*MET*) represents expenditure of energy on activity compared with resting state

### Association of exercise prior to presentation with clinical outcomes

**Fig. 1:**
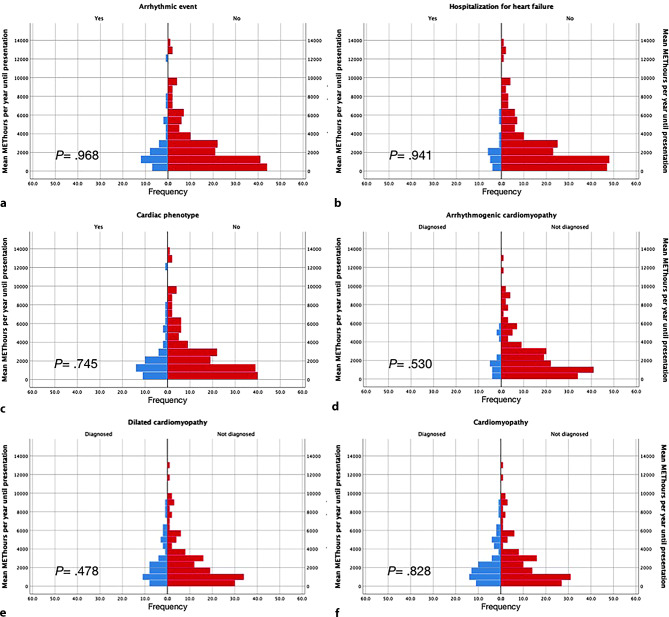
Mean MET-hours per year until presentation in individuals with and without a clinical outcome. Individuals who reached a clinical outcome are depicted in blue, whereas individuals who did not reach a clinical outcome are depicted in *red*. **a** Arrhythmic event (sustained VA), **b** hospitalisation for heart failure, **c** any cardiac phenotype, **d** diagnosis of arrhythmogenic right ventricular cardiomyopathy, **e** diagnosis of dilated cardiomyopathy and **f** diagnosis of any cardiomyopathy

Figure 1 shows median MET-hours/year before presentation in individuals with and without: (a) a sustained VA, (b) HF hospitalisation, (c) any cardiac phenotype during follow-up (including sustained VA, HF, ARVC or DCM diagnosis), (d) ARVC diagnosis, (e) DCM diagnosis and (f) any cardiomyopathy diagnosis at last follow-up. For all outcomes, there was no significant difference in amount of exercise (MET-hours/year) prior to presentation. Tab. 1 shows that there were no differences in clinical outcome when comparing individuals participating in the 50% higher mean MET-hours/year with those in the lower 50% mean MET-hours/year until presentation. This includes a similar frequency of sustained VA (18.3% vs 18.4%; *p* = 0.974) and hospitalisation for HF at last follow-up (9.6% vs 8.7%; *p* = 0.827). There were also no differences in clinical outcome characteristics when comparing individuals from the 25% highest mean MET-hours/year with those in the 25% lowest mean MET-hours/year until presentation (data not shown). Figure S1 in the Electronic Supplementary Material shows similar results when looking only at the median MET-hours/year spent on leisure activities, including sports.

Thirty-eight individuals (18.4%) had experienced a sustained VA at last follow-up, including 11.6% (*n* = 24) with a sustained VT/VF and 6.8% (*n* = 14) with appropriate ICD therapy. We also asked participants on the circumstances of their first arrhythmia. Almost half (*n* = 12/25; 48%) were physically active (e.g. bicycling, gardening).

### Association of exercise prior to presentation with clinical outcomes during follow-up

**Fig. 2:**
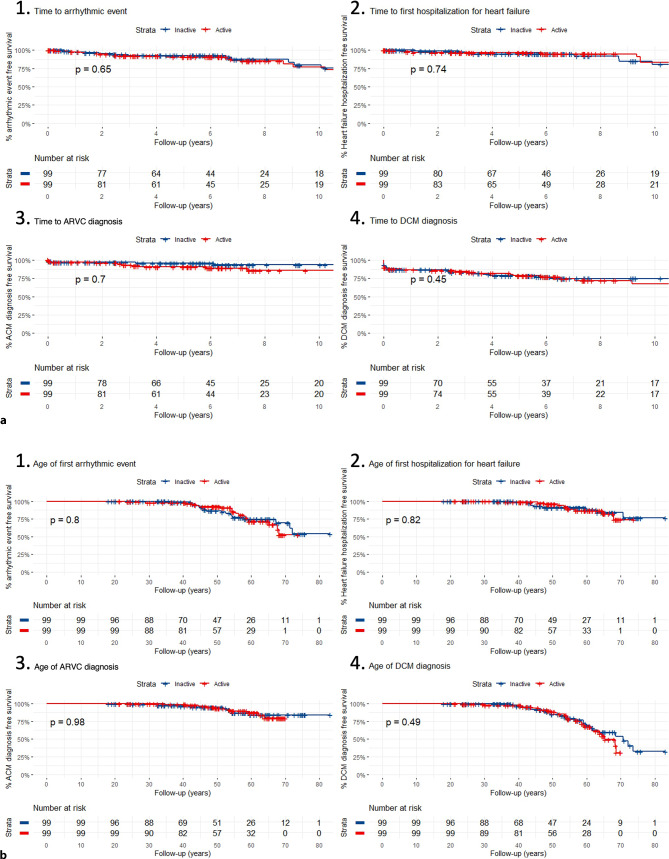
Kaplan-Meier curves of **a** clinical outcomes during follow-up and **b** age of clinical outcomes, stratified by level of activeness. *Red line* depicts active individuals: those who participated in 50% highest mean MET-hours per year until presentation. *Blue line* reflects the inactive group: individuals who participated in 50% lowest mean MET-hours per year until presentation. With (*1*) arrhythmic event (sustained ventricular arrhythmias), (*2*) hospitalisation for heart failure, (*3*) diagnosis of arrhythmogenic right ventricular cardiomyopathy (*ARVC*), and (*4*) diagnosis of dilated cardiomyopathy (*DCM*)

We then looked at survival free from clinical outcomes during follow-up. Nine patients were excluded due to insufficient duration of follow-up. Tab. 3 shows the baseline characteristics of this subpopulation, and Table S1 in the Electronic Supplementary Material lists the univariate and multivariate analyses of survival free from VA and HF hospitalisation. There was no difference between the active and inactive groups in survival free from new sustained VA or HF hospitalisation during follow-up (Fig. 2a, panels 1 and 2) or from diagnosis of ARVC or DCM (baseline or follow-up) (Fig. 2a, panels 3 and 4). There was also no difference between the quartiles of activity in survival free from diagnosis of ARVC or DCM (baseline or follow-up) or new sustained VA or hospitalisation for HF during follow-up (see Figures S2 and S3 in Electronic Supplementary Material).

**Table 3:** Baseline characteristics of 198 carriers with additional clinical follow-up

Characteristic	Total (*N* = 198)	Active (*n* = 99)	Inactive (*n* = 99)	*P*-value
Male	79 (39.9)	46 (46.5)	33 (33.3)	0.059
Age at presentation, years	44	43	45	0.228
Non-sustained VT	33 (16.7)	17 (17.2)	16 (16.2)	0.849
Negative T wave in precordial leads (V1–V6)	33/159 (20.8)	17/79 (21.5)	16/80 (22.2)	0.813
Negative T wave in inferior leads (II, III, aVF)	18/159 (11.3)	11/79 (13.9)	7/80 (8.8)	0.303
Microvoltages on ECG	34/161 (21.1)	16/80 (20)	18/81 (22.2)	0.730
RVEF < 45%	23/138 (16.7)	16/69 (27.1)	7/69 (10.1)	0.07
LVEF < 45%	33/140 (23.6)	19/70 (27.1)	14/70 (20)	0.412
PVC count > 500	27/76 (35.5)	14/35 (40)	13/41 (31.7)	0.446

Data are *n* (%) or mean

*VT* ventricular tachycardia, *ECG* electrocardiogram, *RVEF* right ventricular ejection fraction, *LVEF* left ventricular ejection fraction, *PVC* premature ventricular contraction

^a^Active group consisted of individuals who participated in 50% highest mean metabolic equivalent of task (*MET*) hours per year until presentation

^b^Inactive group consisted of individuals who participated in 50% lowest mean MET-hours per year until presentation

Next, we used Cox regression with a Firth correction for rare outcomes to assess the independent effect of exercise (MET-hours/year) controlling for clinical risk factors on survival free from sustained VA or HF hospitalisation (see Table S1 in Electronic Supplementary Material). Model 1 showed that when controlling for sex and age of presentation, there was no association with the amount of exercise (MET-hours/year) and either sustained VA or HF hospitalisation. Model 2, which includes clinical covariates, likewise showed that sustained VA and HF hospitalisation were not associated with the amount of exercise. Restricted cubic splines showed non-linearity, with model 1 showing *p* = 0.242 for HF hospitalisation and *p* = 0.177 for VA and model 2 showing *p* = 0.308 for HF hospitalisation and *p* = 0.391 for VA (see Figure S4 in Electronic Supplementary Material). The relationship between mean MET-hours/year until presentation and all outcomes showed in all curves a bell-shaped relationship with the top right after the median of 1660 mean MET-hours/year until presentation.

### Penetrance

Figure 2b and S3 (in Electronic Supplementary Material) show the association between exercise and life-time penetrance when split by median MET-hours/year and quartiles of MET-hours/year respectively. These figures highlight that the amount of exercise prior to presentation had no effect on the penetrance of an arrhythmic phenotype, hospitalisation for HF or ARVC/DCM diagnosis.

### Exercise after presentation

Most participants (64.7%) decreased their annual exercise duration and intensity after first presentation (Tab. 2). This was significant for both MET-hours and for total hours spent on any activity (*p* < 0.001). More than half (*n* = 131; 63.2%) of the study population decreased their MET-hours after presentation, and most also decreased their time spent on being active (*n* = 127; 61.4%). In contrast, 63 individuals (30.4%) increased their level of activity in MET-hours/year and 68 (32.9%) individuals increased their time spent on activity.

Exercise restriction is advised for ARVC patients and those with a (likely) pathogenic desmosomal gene variant and this advice is generally also given to *PLN* p.(Arg14del) carriers with ARVC. To assess the independent effect of decreasing exercise after presentation controlling for the same risk factors as previously, we used Cox regression. The univariable and multivariable analyses (see Table S2 in Electronic Supplementary Material) show similar results as presented in supplementary Tab. 1. After controlling for covariates, both model 1 and 2 showed no association with decreasing amount of exercise after presentation and either sustained VA or HF.

## Discussion

This study assessed the effect of the amount of exercise as measured by MET-hours/year on penetrance and clinical outcomes in individuals with *PLN* p.(Arg14del). Our results suggest exercise history prior to clinical presentation does not predict development of an arrhythmic phenotype or HF in this cohort that was only moderately active. These results are in contrast with studies of ARVC patients who generally have (likely) pathogenic desmosomal gene variants.

### Prior studies on influence of exercise on development of ARVC

The relationship between athlete status and the ARVC-phenotype has been studied extensively. Currently, both definite ARVC patients and those with a (likely) pathogenic variant in a desmosomal gene, are discouraged from competitive or frequent high-intensity endurance exercise [8].

An association between exercise and the risk of VA, HF and diagnosis of ARVC in desmosomal variant carriers has been established by multiple studies [9, 24]. In addition, for *TMEM43* p.(Ser358Leu) related ARVC, ≥ 9.0 MET-hours/day in the year prior to ICD implantation was associated with an 9.1-fold increased hazard of first appropriate ICD discharge [25]. Moreover, even in gene-elusive ARVC patients exercise has been shown to have a negative effect by modifying cardiac structure and promoting arrhythmias [26]. However, exercise in *Dsp* haploinsufficient mice partially restored dysregulated gene expression without changing cardiac function, showing that the detrimental effect of exercise is not that straightforward [27].

### Impact of exercise on clinical course of PLN variant carriers

For a yet unaffected, asymptomatic, *PLN* p.(Arg14del) carrier, data from the current study strongly suggest there is no reason to limit exercise if this is of limited-moderate intensity (i.e. 6 METs or less) [8]. This contrasts with desmosomal gene carriers and might be explained by the fact that this *PLN* cohort was not particularly active or that different pathophysiological mechanisms eventually lead to disease. For unaffected asymptomatic *PLN* p.(Arg14del) carriers that want to exercise more intensively, and thereby fulfil the definition of an athlete (≥ 18 MET-hours/week), the uncertainty of an effect of more intensive exercise has to be discussed and participation should be a shared decision.

For an affected individual, the data also suggest that mild-moderate exercise as reflected by the number of MET-hours does not negatively influence disease course. However, it has been observed that incident malignant arrhythmias in *PLN* p.(Arg14del) are often associated with exercise. Previously, it was shown that 74% of malignant VA in *PLN* p.(Arg14del) variant carriers occurred during or shortly after exercise, independent of the intensity [7]. Additionally, out of 19 individuals of whom the circumstances of SCD were known, 13 (68%) were exercising. Similarly in our cohort, those who experienced a VA and could recall their activity at their first arrhythmia, almost half (*n* = 12/25; 48%) said they were active. Thus, for the patient already displaying risk factors related to the development of an arrhythmia (decreased LVEF, a prior sustained or non-sustained VT, low-voltage ECG, the number of leads with negative T waves or PVC count), and for those fulfilling 2010 Task Force Criteria for ARVC [18], it is reasonable to discuss exercise restrictions and make a shared decision on the intensity of participation [8]. In short, it appears that exercise can be an arrhythmic trigger in *PLN* cardiomyopathy patients but is likely not associated with disease development or progression.

### Study limitations

Recall bias and social desirability bias were limiting factors, as exercise history was assessed retrospectively. A potential future prospective study on exercise in a large number of phenotype negative *PLN* p.(Arg14del) carriers would be ideal to study the effect of exercise on disease penetrance. By using the same methods as previous studies of exercise in ARVC patients, we tried to minimise the aforementioned limiting factors. Mortality was not available as an outcome measure, as patients had to be alive to participate in the interview, which could lead to underrepresentation of patients with worse outcomes. On the other hand, we recruited patients from tertiary care centres, who commonly have more severe disease and worse outcomes. Last, even though we had a response rate of 58.6%, voluntary response bias could have played a role.

In addition, the relatively small study population that was not particularly active may hamper generalisation of these findings to more intensive levels of activity and providing advice to high level athletes with this *PLN* variant.

## Conclusion

There was no association between exercise (MET-hours/year) and lifetime disease penetrance, sustained VA or HF hospitalisation during follow-up in mild-moderately active *PLN* p.(Arg14del) carriers. This seems to contrast with prior studies in ARVC patients and desmosomal variant carriers. In addition, diagnosis of ARVC or DCM was not associated with mild-moderate exercise. Exercise restrictions are not advised for mild-moderately active (those not participating in frequent or competitive or endurance exercise (> 6 METs)) *PLN* p.(Arg14del) carriers without any known risk factors for arrhythmias. However, *PLN* p.(Arg14del) carriers with risk-factors may benefit from exercise restriction because arrhythmias are associated with exercise in nearly half of the events.

## Supplementary Information

**Table S1** Hazard ratios for sustained ventricular arrhythmias or heart failure according to exercise history and clinical characteristics for 198 individuals

**Table S2** Hazard ratios for arrhythmic event or heart failure according to increasing activity after presentation and clinical characteristics

**Figure S1** Mean MET-hours per year for leisure activities until presentation in individuals with and without an event or diagnosis

**Figure S2** Kaplan-Meier curves of event or diagnosis during follow-up stratified by level of activeness (quartiles)

**Figure S3** Kaplan-Meier curves of age of event or diagnosis stratified by level of activeness (quartiles)

**Figure S4 **Restricted cubic splines showing non-linearity. Mean MET-hours/year until presentation and all outcomes show a bell-shaped relationship with the top right after the median of 1660 mean MET-hours/year until presentation. Model 1 showing **a** *p* = 0.242 for HF hospitalisation and **b** *p* = 0.177 for VA. Model 2 showing **c*** p* = 0.308 for HF hospitalisation and **d** *p* = 0.391 for VA
