# Supplementary material for: Exercise does not influence development of phenotype in PLN p.(Arg14del) cardiomyopathy
Source: Neth Heart J. 2023 Jul 20;31(7-8):291–9. doi: 10.1007/s12471-023-01800-4 (PMC10400740; doi:10.1007/s12471-023-01800-4)
Supplement: Supplementary file 1 — Table S1 Hazard ratios for sustained ventricular arrhythmias or heart failure according to exercise history and clinical characteristics for 198 individuals [file 12471_2023_1800_MOESM1_ESM.docx]

**Table S1** Hazard ratios for sustained ventricular arrhythmias or heart failure according to exercise history and clinical characteristics for 198 individuals

| **Variable** | **VT/VF** | | | **Heart failure hospitalization** | | |
| --- | --- | --- | --- | --- | --- | --- |
|  | Unadjusted | Model 1 | Model 2 | Unadjusted | Model 1 | Model 2 |
|  | HR (95%CI) | HR (95%CI) | HR (95%CI) | HR (95% CI) | HR (95%CI) | HR (95%CI) |
| Active | 1.19 (0.56-2.50) | 1.12 (0.54-2.36) | 1.02 (0.45-2.33) (0.57-4.28) | 1.18 (0.48-2.93) | 1.11 (0.45-2.75) | 1.68 (0.61-4.85) |
| Male | 1.26 (0.60-2.65) |  |  | 0.68 (0.27-1.74) |  |  |
| Age at presentation | 1.03 (1.00-1.06) |  |  | 1.04 (1.00-1.08) |  |  |
| RVEF <45% | 3.08 (1.16-8.19)* |  |  | 18.87 (4.89-72.91)* |  |  |
| LVEF <45% | 6.46 (2.59-16.1)* |  |  | 12.73 (3.49-46.46)* |  |  |
| Amount of negative T leads | 1.31 (1.10-1.54)* |  |  | 0.83 (0.59-1.17) |  |  |
| Microvoltages on ECG | 4.23 (1.57-11.37)* |  |  | 6.07 (1.92-19.25)* |  |  |
| Sustained VT/VF at presentation | 2.72 (1.02-7.27)* |  |  | 1.39 (0.40-4.88) |  |  |
| PVC-count > 500 | 8.41 (0.98-72.50) |  |  | ^ |  |  |
| NSVT | 3.4 (1.61-7.21)* |  |  | 3.31 (1.34-8.19)* |  |  |

Model 1 contains sex and age at presentation. Model 2 contains all covariates. CI indicates confidence interval; LVEF, left ventricular ejection fraction; HR,

hazard ratio; NSVT, non-sustained ventricular tachycardia; PVC, premature ventricular complexes; RVEF, right ventricular ejection fraction; VT, ventricular

tachycardia; VF, ventricular fibrillation. * represents statistically significant hazard ratios with a p-value <0.05. ^ contains fields where missing data was too extensive for a

reliable estimate
