# Supplementary material for: Exercise does not influence development of phenotype in PLN p.(Arg14del) cardiomyopathy
Source: Neth Heart J. 2023 Jul 20;31(7-8):291–9. doi: 10.1007/s12471-023-01800-4 (PMC10400740; doi:10.1007/s12471-023-01800-4)
Supplement: Supplementary file 2 — Table S2 Hazard ratios for arrhythmic event or heart failure according to increasing activity after presentation and clinical characteristics [file 12471_2023_1800_MOESM2_ESM.docx]

**Table S2** Hazard ratios for arrhythmic event or heart failure according to increasing activity after presentation and clinical characteristics

| **Variable** | **VT/VF** | | | **Heart failure hospitalization** | | |
| --- | --- | --- | --- | --- | --- | --- |
|  | Unadjusted | Model 1 | Model 2 | Unadjusted | Model 1 | Model 2 |
|  | HR (95%CI) | HR (95%CI) | HR (95%CI) | HR (95% CI) | HR (95%CI) | HR (95%CI) |
| More active in follow-up | 0.68 (0.30-1.55) | 1.17 (0.54-2.42) | 1.91 (0.83-4.31) | 1.01 (0.40-2.58) | 1.31 (0.45-3.56) | 0.91 (0.33-2.26) |
| Male | 1.26 (0.60-2.65) |  |  | 0.68 (0.27-1.74) |  |  |
| Age at presentation | 1.03 (1.00-1.06) |  |  | 1.04 (1.00-1.08) |  |  |
| RVEF <45% | 3.08 (1.16-8.19)* |  |  | 18.87 (4.89-72.91)* |  |  |
| LVEF <45% | 6.46 (2.59-16.1)* |  |  | 12.73 (3.49-46.46)* |  |  |
| Amount of negative T leads | 1.31 (1.10-1.54)* |  |  | 0.83 (0.59-1.17) |  |  |
| Microvoltages on ECG | 4.23 (1.57-11.37)* |  |  | 6.07 (1.92-19.25)* |  |  |
| Sustained VT/VF at presentation | 2.72 (1.02-7.27)* |  |  | 1.39 (0.40-4.88) |  |  |
| PVC-count > 500 | 8.41 (0.98-72.50) |  |  | X |  |  |
| NSVT | 3.40 (1.61-7.21)* |  |  | 3.31 (1.34-8.19)* |  |  |

Model 1 contains sex and age at presentation. Model 2 contains all covariates. CI indicates confidence interval; LVEF, left ventricular ejection fraction; HR, hazard ratio; NSVT, non-sustained ventricular tachycardia; PVC, premature ventricular complexes; RVEF, right ventricular ejection fraction; VT, ventricular tachycardia; VF, ventricular fibrillation.
