# Supplementary figures and images for: Exercise does not influence development of phenotype in PLN p.(Arg14del) cardiomyopathy
Source: Neth Heart J. 2023 Jul 20;31(7-8):291–9. doi: 10.1007/s12471-023-01800-4 (PMC10400740; doi:10.1007/s12471-023-01800-4)

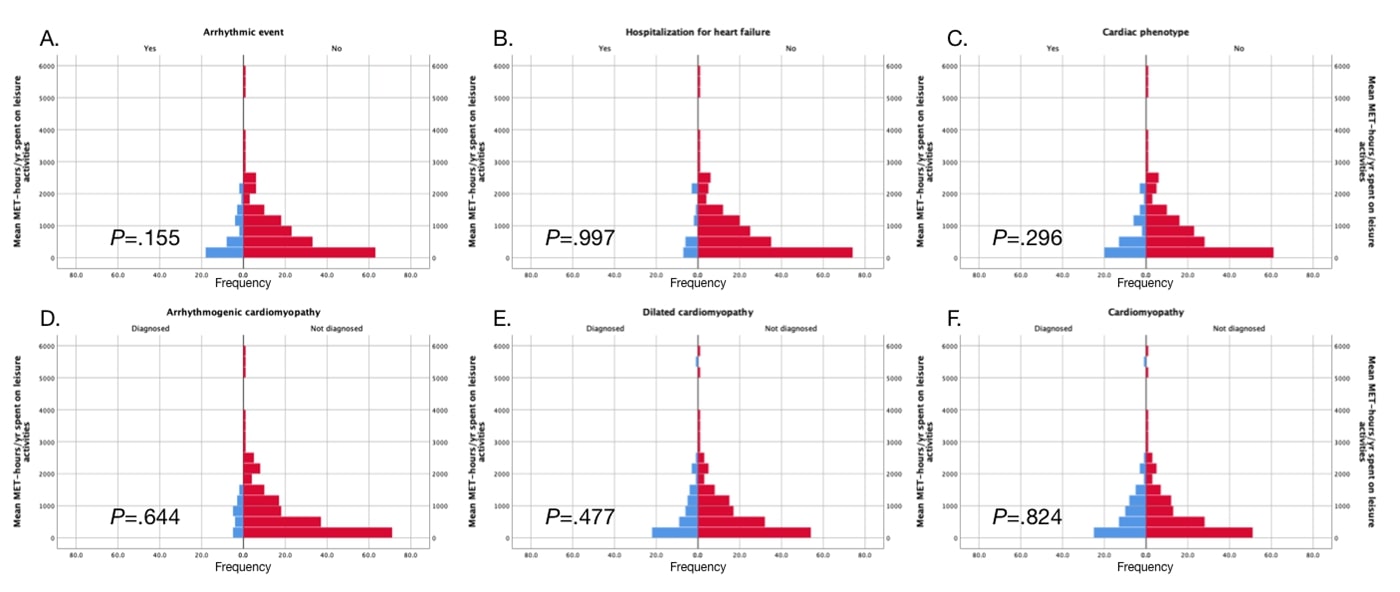

Supplement: Supplementary file 3 — Figure S1 Mean MET-hours per year for leisure activities until presentation in individuals with and without an event or diagnosis [file 12471_2023_1800_MOESM3_ESM.jpg]

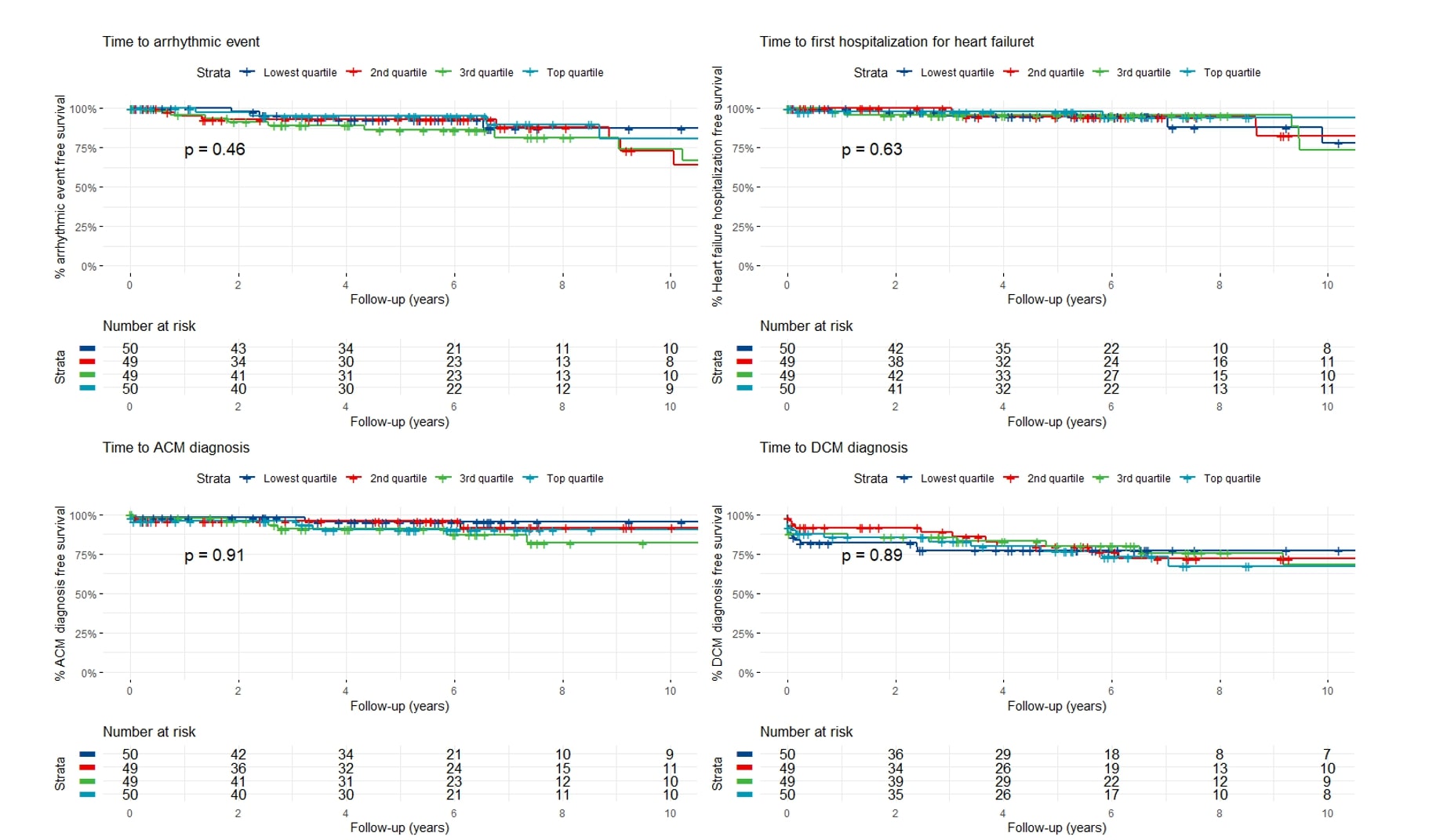

Supplement: Supplementary file 4 — Figure S2 Kaplan-Meier curves of event or diagnosis during follow-up stratified by level of activeness (quartiles) [file 12471_2023_1800_MOESM4_ESM.jpg]

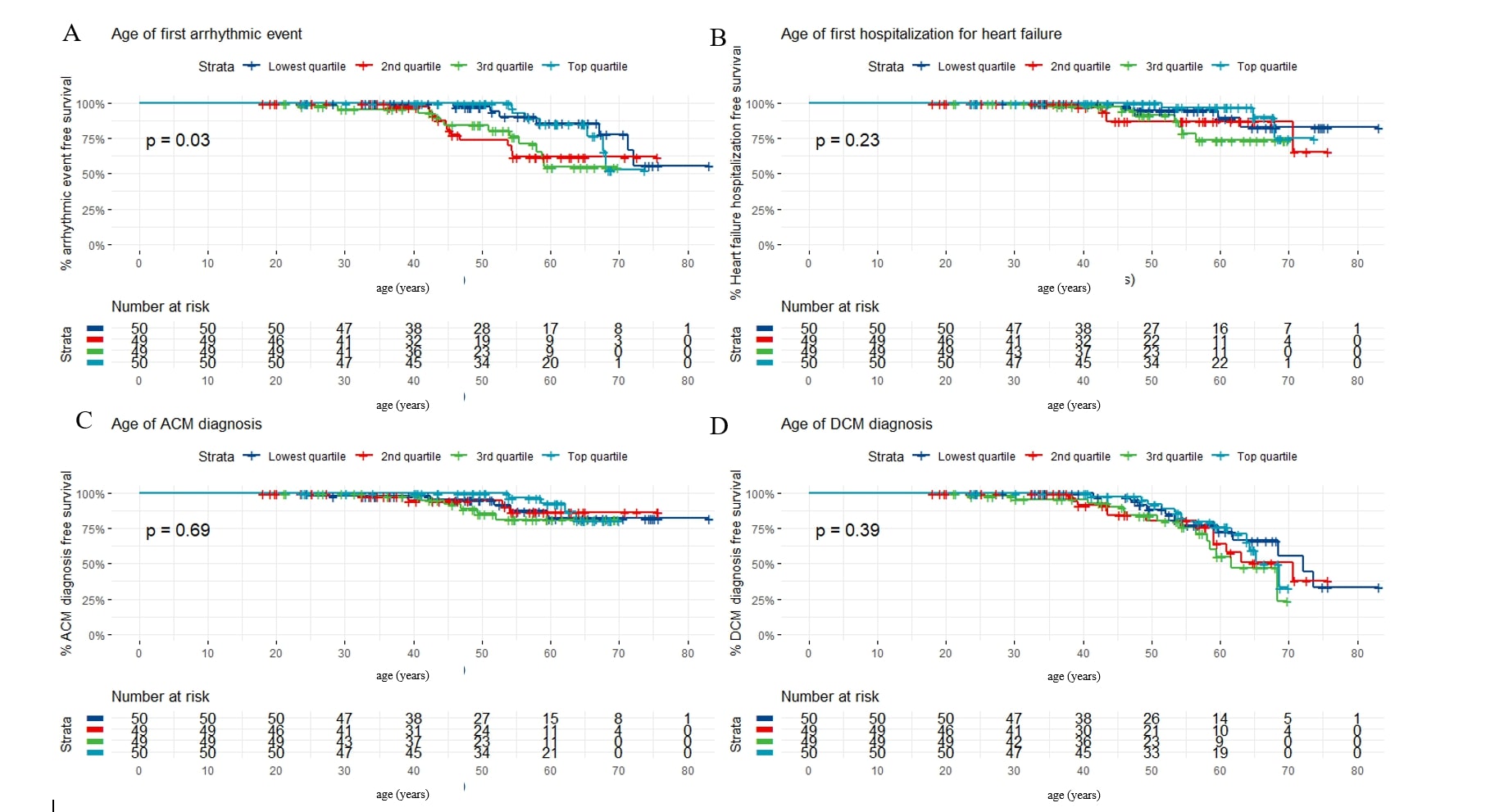

Supplement: Supplementary file 5 — Figure S3 Kaplan-Meier curves of age of event or diagnosis stratified by level of activeness (quartiles) [file 12471_2023_1800_MOESM5_ESM.jpg]

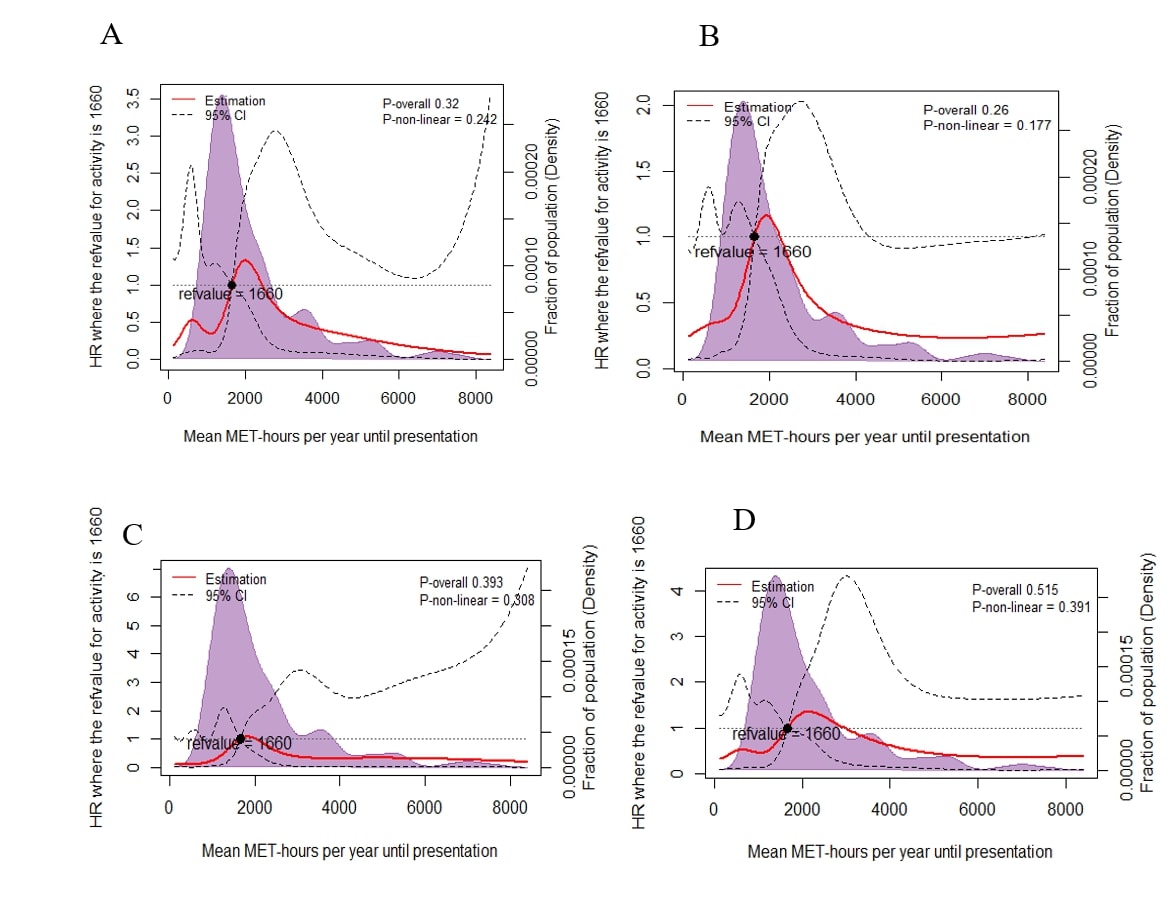

Supplement: Supplementary file 6 — Figure S4 Restricted cubic splines showing non-linearity. Mean MET-hours/year until presentation and all outcomes show a bell-shaped relationship with the top right after the median of 1660 mean MET-hours/year until presentation. Model 1 showing a p = 0.242 for HF hospitalisation and b p = 0.177 for VA. Model 2 showing c p = 0.308 for HF hospitalisation and d p = 0.391 for VA [file 12471_2023_1800_MOESM6_ESM.jpg]
